# Depth‐to‐Areal Staging Heterogeneity Preceding Lithium Plating in Graphite Anodes Revealed by Operando Imaging

**DOI:** 10.1002/advs.77918

**Published:** 2026-09-27

**Authors:** Tae Young Choi, Juyoung Kim, Vithiya Muralidharan, Seungwoo Choi, Jeongwoo Seo, Seungwoo Ryu, Myeongjun Choi, Hyun‐Wook Lee

**Affiliations:** ^1^ School of Energy and Chemical Engineering Ulsan National Institute of Science and Technology (UNIST) Ulsan Republic of Korea

**Keywords:** areal heterogeneity, depth heterogeneity, electrode porosity, graphite staging, lithium plating, operando imaging

## Abstract

Lithiation in graphite electrodes proceeds heterogeneously, yet how this heterogeneity develops in space, and how it responds to the kinetic conditions of charging, remains poorly resolved because conventional probes report only volume‐averaged behavior. Here we resolve how graphite staging heterogeneity evolves across both the through‐thickness and in‐plane directions of the electrode, and how its severity is governed by charging kinetics. Combining operando optical microscopy, in situ x‐ray diffraction, color‐space analysis, and electrode‐scale simulation, we track lithiation from the particle scale to the full electrode as a function of electrode porosity and charging rate. Lower porosity and higher rate intensify tortuosity‐controlled Li^+^ transport limitation, generating through‐thickness lithiation gradients that evolve into pronounced in‐plane staging non‐uniformity, such that neighboring regions advance through the staging sequence at markedly different rates. Critically, operando imaging directly reveals that domains reaching advanced lithiation states earliest become the preferential sites at which lithium plating initiates, demonstrating that plating is spatially templated by pre‐existing staging heterogeneity rather than nucleating randomly. These findings establish staging heterogeneity as a kinetically controlled precursor to plating and provide a general operando framework for diagnosing heterogeneity‐driven behaviour in insertion electrodes.

## Graphical abstract

Fast charging drives graphite anodes far from equilibrium, yet where lithium plating begins has remained hidden. Operando optical microscopy here reveals that neighboring regions advance through the staging sequence asynchronously, and that domains reaching advanced lithiation earliest become the preferential sites where plating initiates. Electrode porosity governs this heterogeneity, identifying architecture as a lever against plating.

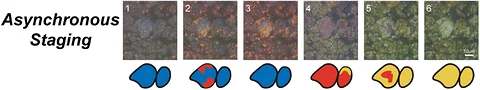

## 1 Introduction

The pursuit of fast‐charging lithium‐ion batteries has intensified interest in the electrochemical processes that govern electrode behavior under dynamic operating conditions. As charging rates increase, electrochemical reactions are driven progressively further from equilibrium, producing non‐uniform lithium distribution, elevated polarization, and accelerated degradation [1, 2, 3]. Within practical porous electrodes, lithiation is therefore spatially heterogeneous rather than uniform: the coupled effects of ionic transport limitations, electronic conduction pathways, interfacial reaction kinetics, and phase‐transition dynamics generate pronounced local variations in reaction rate, lithium concentration, and thermodynamic state [4, 5]. Because these heterogeneities strongly influence electrode utilization, rate capability, and degradation behavior, and ultimately battery lifetime, resolving their origin and evolution during fast charging has become a central objective of contemporary battery research [2, 6, 7, 8].

Reaction heterogeneity spans a wide range of spatial and temporal scales, from atomic‐scale lithium distribution, through particle‐level phase transformations, to electrode‐scale concentration gradients [1, 4, 5, 9, 10, 11]. Under fast‐charging conditions, electrode‐scale heterogeneity becomes particularly consequential because it directly reflects the interplay between electrode architecture and ionic transport: as charging proceeds, local differences in lithium‐ion availability establish non‐uniform lithiation pathways and, with them, spatial variations in state of charge, phase evolution, and electrode utilization [12, 13]. Graphite is an especially informative system in which to resolve this behavior, because its lithiation proceeds through a sequence of well‐defined staging transitions, each associated with a distinct lithium concentration [13, 14, 15, 16, 17]. Graphite staging thus acts as a direct, spatially resolved reporter of local reaction progress, and the distribution of stages encodes how lithiation propagates through a porous electrode under non‐equilibrium conditions.

The severity of this heterogeneity is closely tied to transport limitations within the porous electrode. Structural parameters such as thickness, tortuosity, and porosity strongly influence ionic transport and thereby reshape the spatial distribution of graphite lithiation states [10, 12, 13, 17, 18]. As heterogeneity develops, localized domains reach advanced staging states earlier than their surroundings, and such domains have long been proposed as preferential sites for lithium plating [6, 19, 20, 21, 22]. Direct experimental evidence connecting graphite staging heterogeneity to the onset of plating nonetheless remains scarce, and the spatial and temporal evolution of staging that precedes plating has never been directly visualized. As a result, a fundamental question remains unresolved: whether, and how, pre‐existing staging heterogeneity determines where lithium plating first nucleates.

A central obstacle to answering this question lies in the averaging inherent to conventional characterization. Electrochemical analyses, including voltage profiles, differential capacity, and impedance spectroscopy, capture overall reaction kinetics but average the response over the entire electrode volume [5, 10]. Operando x‐ray diffraction (XRD) resolves graphite phase transitions directly but likewise reports volume‐averaged structural information. These approaches can establish that reaction heterogeneity exists, yet they cannot reveal where it originates, how it evolves spatially, or whether particular staging behaviors precede lithium plating. Operando optical microscopy (operando‐OM) is uniquely suited to close this gap: because graphite displays distinct optical signatures across its staging sequence, operando‐OM visualizes staging evolution with simultaneous spatial and temporal resolution [13, 16, 17], allowing heterogeneous lithiation to be tracked in real time and correlated directly with the location of plating. Even so, how graphite staging heterogeneity develops across both the through‐thickness (depth) and in‐plane (areal) directions of the electrode, and how this multidimensional heterogeneity governs plating, has not been systematically resolved.

Herein, we resolve the origin and evolution of graphite staging heterogeneity during elevated‐rate charging and establish a direct link between this heterogeneity and lithium plating. Combining electrochemical characterization, operando‐OM, in situ XRD, quantitative color‐space analysis, and electrode‐scale simulation, we first show that electrode porosity governs rate‐dependent behavior by setting the tortuosity‐controlled Li^+^ transport and overpotential that develop through the electrode depth. Operando imaging then reveals pronounced in‐plane (areal) staging non‐uniformity under elevated‐rate charging, which develops together with the through‐thickness (depth) gradients imposed by transport, establishing the depth‐to‐areal staging heterogeneity central to this work. Most importantly, we directly observe that lithium plating preferentially nucleates in regions where advanced graphite stages emerge earliest, showing that the location of plating is templated by pre‐existing staging heterogeneity rather than being spatially random. By connecting electrode porosity to depth‐to‐areal staging heterogeneity and, in turn, to plating onset, these findings identify electrode architecture as a direct design lever for suppressing lithium plating in elevated‐rate charging graphite anodes.

## 2 Results and Discussion

### 2.1 Porosity and Rate‐Dependent Staging Heterogeneity

**FIGURE 1:**
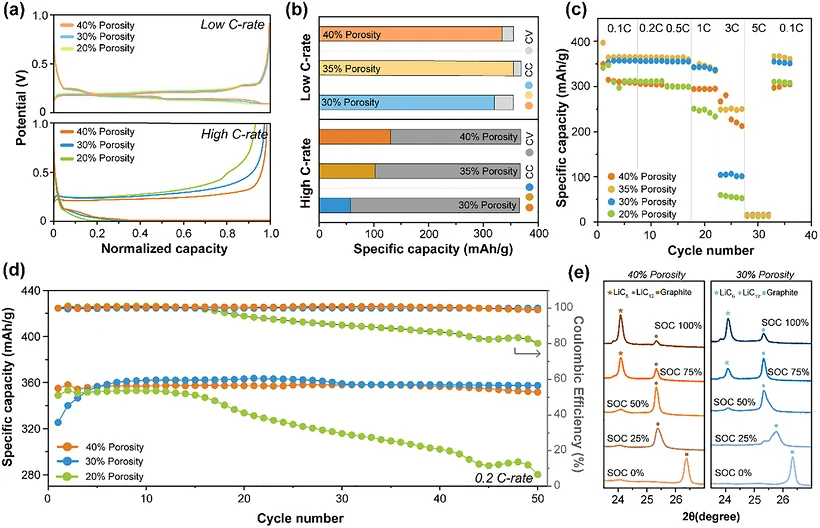
Porosity‐dependent electrochemical behavior and staging evolution of graphite electrodes. (a) Galvanostatic charge‐discharge profiles of graphite electrodes with 40%, 30%, and 20% porosity at 0.1C (low C‐rate; top) and 1C (high C‐rate; bottom), showing the increasing polarization associated with decreasing porosity. (b) Comparison of the specific capacity contributions from the constant‐current (CC; colored regions) and constant‐voltage (CV; grey regions) charging steps under low and high C‐rate conditions for electrodes with different porosities. (c) Rate capability of graphite electrodes with different porosities at 0.1C, 0.2C, 0.5C, 1C, 3C, and 5C, followed by recovery at 0.1C (d) Long‐term cycling performance at 0.2C for the graphite electrodes with 40%, 30% and 20% porosity, showing the specific capacity (left) and corresponding coulombic efficiency (right). (e) In ‐situ XRD patterns of 40% and 30% porosity graphite electrodes at state‐of‐charge (SOC) values of 0%, 25%, 50%, 75%, and 100%, showing the evolution of the graphite, LiC_12_, and LiC_6_ phases during lithiation.

Graphite electrodes with varying porosities (40%, 35%, 30%, and 20%) were fabricated through roll pressing, and their actual porosity values and microstructural characteristics were experimentally measured using Mercury Intrusion Porosimetry and cross‐sectional SEM analysis (Table S1 and Figure S1). These electrodes were assembled into half‐cells and evaluated by galvanostatic charge‐discharge and rate‐capability measurements to determine how electrode architecture governs rate‐dependent behavior. The voltage profiles exhibited increasingly pronounced polarization as the charging rate increased (Figure 1a and Figure S2). At a low charging rate, all electrodes delivered nearly identical voltage profiles, indicating minimal transport limitation. Under a high charging rate, however, clear differences emerged. The 30% porosity electrode reached the cut‐off voltage earlier and developed substantially larger polarization than the 40% porosity electrode, reflecting more severe transport limitation during lithiation. Conversely, the 40% porosity electrode delivered a slightly lower capacity under low‐rate conditions, which may arise from its reduced packing density and the less effective interparticle electronic contact associated with a highly porous structure. Its superior performance under elevated‐rate charging nonetheless demonstrates that electrode porosity becomes a decisive factor governing reaction behavior once transport limitations become significant.

To quantify the influence of porosity, the specific capacities obtained under low‐ and high‐rate charging are summarized in Figure 1b. Under low‐rate operation, all electrodes delivered comparable capacities. Increasing the charging rate, however, sharply reduced the capacity of the lower‐porosity electrodes, whereas the 40% porosity electrode retained a considerably larger fraction of its theoretical capacity. Higher porosity therefore mitigates polarization by facilitating ionic transport through the porous electrode network, and this divergence indicates that porosity becomes increasingly important as transport limitations begin to dominate lithiation at higher rates. Rate‐capability measurements from 0.1 to 5C, followed by recovery at 0.1C, further resolved this behavior (Figure 1c). At 0.1C to 0.5C, all electrodes exhibited comparable capacities, indicating relatively homogeneous lithiation. As the rate increased to 1C and above, capacity retention decreased progressively, with the divergence becoming the most pronounced at 3C and 5C, reflecting the onset of severe rate‐dependent transport limitations and elevated overpotentials across the electrode network [2, 23]. When the rate returned to 0.1C, all electrodes largely recovered their initial capacities, indicating that high‐rate capacity loss originated primarily from reversible kinetic and transport limitations rather than irreversible degradation. Although macroscopic capacity measurements capture only volume‐averaged electrochemical responses, the underlying spatial staging heterogeneity is directly resolved via operando‐OM and in situ XRD in subsequent sections. Because 1C marks the onset where porosity‐dependent behavior becomes clearly distinguishable while sustaining staging transitions at a timescale suitable for operando imaging, it was selected as the representative elevated‐rate charging condition [24, 25, 26]. At the same time, 1C sustains staging transitions slowly enough to be tracked with the temporal resolution of operando imaging and in situ XRD, whereas at higher rates the transitions proceed too rapidly for individual staging states to be reliably resolved. This rate therefore isolates the intrinsic porosity‐driven heterogeneity while keeping the staging evolution experimentally observable.

Electrode porosity also governs long‐term electrochemical stability. Long‐term cycling at 0.2C (Figure 1d) showed continuous capacity fading for the 20% porosity electrode, whereas the 30% and 40% porosity electrodes maintained relatively stable capacities over the same period. The progressive capacity loss of the low‐porosity electrode reflects reduced active‐material utilization arising from persistent ionic transport limitation and increased polarization during repeated lithiation and delithiation. To further examine the evolution of cycling behavior, additional cycling data were analyzed together with the contribution of the constant‐voltage (CV) charging step, which was quantified as the CV step‐capacity ratio (Figure S3). The 40% porosity electrode maintained a nearly constant ratio throughout cycling, indicating that most lithiation proceeded during constant‐current (CC) charging. The 30% porosity electrode, by contrast, showed a progressively increasing ratio, particularly on prolonged cycling, indicating that an increasing fraction of the total capacity was delivered during the CV stage. Decreasing porosity therefore drives the cell into the CV region progressively earlier as cycling proceeds, reflecting increasingly severe transport limitation and polarization that suppress lithium insertion during CC charging. These combined cycling and CV step‐capacity ratio analyses reveal that, although lower porosity can initially deliver comparable total capacity at mild rates through extended CV compensation (Figure S3a), it progressively suppresses the accessible CC capacity and accelerates long‐term cycling degradation as the lithiation pathway shifts toward CV‐dominated charging (Figure S3b).

To examine how this heterogeneity develops, differential‐capacity (dQ/dV) analysis was performed for all electrodes (Figure S4). As the charging rate increased, the dQ/dV peaks systematically weakened and broadened. Sharp peaks correspond to well‐defined staging transitions confined to a narrow voltage range, whereas peak broadening indicates that nominally identical transitions become distributed over a wider voltage window as a result of spatially non‐uniform local reaction states within the electrode [24]. Two features are notable. First, at identical C‐rates, the lower‐porosity electrodes showed substantially greater broadening and distortion than the higher‐porosity electrode. Second, at 1C several staging‐related peaks in the 30% porosity electrode broadened to the point of being barely distinguishable. These signatures indicate that graphite lithiation proceeds increasingly far from equilibrium as the charging rate rises. Conventional electrochemical measurements alone, however, cannot reveal how these non‐equilibrium reactions evolve spatially within the electrode.

To track graphite phase evolution directly, in situ XRD was performed at 1C as a complement to dQ/dV analysis. Whereas dQ/dV yields only indirect signatures of phase transitions, in situ XRD explicitly assigns graphite staging and correlates specific diffraction peaks with the state of charge (SOC) (Figure 1e and Figure S5) [27]. The 40% porosity electrode exhibited relatively uniform evolution of the staging peaks, whereas the 30% porosity electrode showed broader and more asynchronous changes in peak intensity and position (Figure 1e). More importantly, the relative fractions of the staging phases differed considerably even at identical SOC: although both electrodes received the same charge and reached the same average SOC, their stage compositions were markedly different, showing that lithium was partitioned differently among the available graphite phases. Average SOC alone therefore cannot describe the lithiation state of a graphite electrode under elevated‐rate charging. Correlating the XRD‐derived staging evolution with the electrochemical profiles further shows that the 40% porosity electrode entered the CV region only after roughly half of the total lithiation had been achieved, so that reaction limitations became significant mainly during the later stages of lithiation. The 30% porosity electrode entered the CV regime at a considerably lower SOC, indicating that transport limitations shaped staging evolution across a much broader lithiation range; even at nominal 100% SOC it retained a significant fraction of graphite in earlier stages, indicating incomplete utilization of the active material.

Taken together, these results show that decreasing electrode porosity produces increasingly heterogeneous lithiation by redistributing lithium among the graphite staging phases. In situ XRD identifies this staging evolution directly, but provides only volume‐averaged structural information and therefore cannot resolve how the staging differences are distributed in space. Resolving the spatial origin of graphite staging heterogeneity thus requires direct visualization. Accordingly, operando‐OM was performed on cross‐sectioned graphite electrodes to map the spatial evolution of graphite staging and to establish its relationship with preferential lithium plating during elevated‐rate charging.

### 2.2 Porosity Control of Through‐Thickness Staging Heterogeneity and Plating Onset

Depth‐resolved optical imaging combined with quantitative color analysis allows the evolution of local lithiation states to be monitored directly throughout the electrode thickness. During lithiation, graphite undergoes distinct optical color transformations corresponding to its sequential staging evolution, with Stage 1L represented by a grey/dark phase, Stage 3L by a blue phase, Stage 2 by a red‐orange phase, and Stage 1 by a golden‐yellow phase. Operando‐OM reveals that electrode porosity plays a decisive role in governing through‐thickness lithiation heterogeneity, and that this heterogeneity intensifies under fast charging [13, 16, 17]. Under low‐rate lithiation, the 40% porosity electrode exhibited relatively uniform color evolution across the entire thickness, indicating homogeneous staging progression from the separator to the current collector. The 30% porosity electrode, by contrast, developed noticeable spatial variation as lithiation progressed along the depth direction, characterized by the simultaneous coexistence of a fully lithiated golden‐yellow domain (Stage 1) near the separator face, an intermediate red‐orange phase (Stage 2), and an under‐lithiated blue phase (Stage 3L) towards the current collector (Figure S6). Because these color changes correspond to different graphite staging states, lithiation evidently proceeds less uniformly in the lower‐porosity electrode even under mild conditions.

**FIGURE 2:**
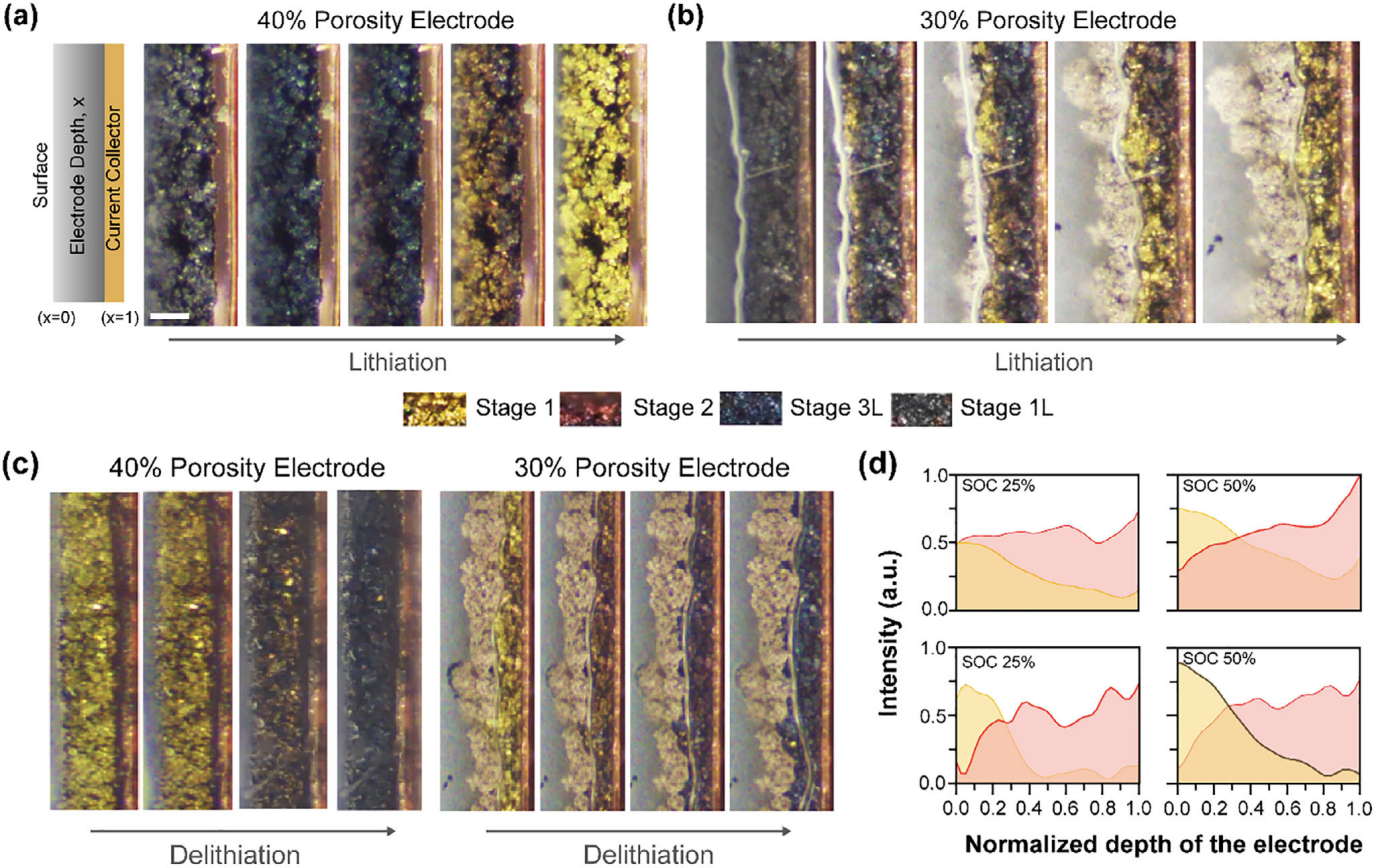
Operando‐OM visualization of depth‐resolved staging evolution and its reversibility. (a, b) Time‐resolved cross‐sectional operando‐OM images showing lithiation progressing through the electrode thickness for (a) 40% and (b) 30% porosity electrodes. The separator is located at the left side (*x* = 0) and the current collector at the right (*x* = 1). The observed optical contrasts correspond to different graphite staging states: golden‐yellow, Stage1; red‐orange, Stage 2; blue, Stage3L; and grey/dark, Stage 1L. (c) Operando‐OM images acquired during delithiation for the 40% and 30% porosities, showing the evolution of the graphite staging states and differences in lithium‐plating morphology after charging. (d) Quantitative through‐thickness staging profiles obtained by CIE (Commission Internationale de l'Éclairage) L*a*b* color‐space analysis at representative SOCs of 25% and 50% for the 40% and 30% porosity electrodes.

This porosity dependence became substantially more pronounced as the charging rate increased. At 1C, the 40% porosity electrode largely maintained coherent staging evolution through the thickness, with the lithiation front advancing relatively uniformly from the separator towards the current collector (Figure 2a). The 30% porosity electrode instead developed strong depth‐dependent phase separation (Figure 2b), where the surface region rapidly reached the fully lithiated golden‐yellow phase (Stage 1), whereas the interior retained a mixed red‐orange phase (Stage 2) and golden‐yellow phase (Stage1) coloration, indicating the coexistence of different lithiation states and increasingly heterogeneous reaction across the thickness. Therefore, the reduced porosity not only promotes lithiation heterogeneity but also amplifies its development under fast‐charging conditions. The emergence of strong through‐thickness gradients was accompanied by lithium plating at the electrode surface. The plating was observed more extensively on the low‐porosity electrodes, which also exhibited the most severe staging heterogeneity, pointing to a correlation between transport‐induced lithiation non‐uniformity and the formation of plating‐prone regions. Notably, plating morphology also varied with both porosity and charging condition, with the morphology of the deposited lithium differing considerably even at identical rates, indicating that the local electrochemical environment strongly shapes plating behavior. This morphology in turn governed the subsequent delithiation (Figure 2c); relatively uniform deposits were stripped more homogeneously during discharge, whereas localized, dendritic deposits showed reduced reversibility and persisted longer [10].

To quantify the observed gradients, the operando‐OM images were analyzed by CIE (Commission Internationale de l'Éclairage) L*a*b* (L* is the lightness, a* is the green to red axis, and b* is the blue to yellow axis) color‐space fitting [13, 17, 28, 29, 30]. For each condition, regions of identical color were sampled at the same reaction time to compare how nominally equivalent optical states distribute along the electrode depth. In the resulting profiles, the horizontal axis denotes the normalized electrode thickness from separator (*x* = 0) to current collector (*x* = 1), and the vertical axis the relative fraction of the fully lithiated golden‐yellow phase extracted from the fitting. A flat profile reflects uniform accumulation of the golden‐yellow phase through the thickness, whereas a steep profile reflects lithiation preferentially localized near specific regions. The analysis in Figure 2d corroborates the operando imaging: the 40% porosity electrode retained comparatively uniform golden‐yellow phase distributions across the thickness, largely preserved even at elevated rates, whereas the 30% porosity electrode consistently showed steeper gradients, with the golden‐yellow phase concentrated near the separator and decreasing sharply towards the interior. The magnitude of this gradient grew systematically with charging rate. Reducing electrode porosity thus produces increasingly severe through‐thickness lithiation heterogeneity, which elevated‐rate charging further amplifies.

Collectively, the operando‐OM results show that transport limitations arising from reduced porosity generate substantial depth‐dependent staging heterogeneity within graphite electrodes. Whereas the electrochemical and in situ XRD measurements in Section 2.1 capture only the volume‐averaged consequences of these processes, operando‐OM resolves the spatial origin and evolution of the heterogeneity directly, linking electrode‐scale transport limitation to local staging evolution along the electrode depth. Having established this through‐thickness (depth) heterogeneity, we next examine how staging non‐uniformity develops in the in‐plane (areal) direction, and how these two combine to template lithium plating.

### 2.3 Transport‐Induced Origin of Through‐Thickness Lithiation Heterogeneity From Electrode‐Scale Simulations

**FIGURE 3:**
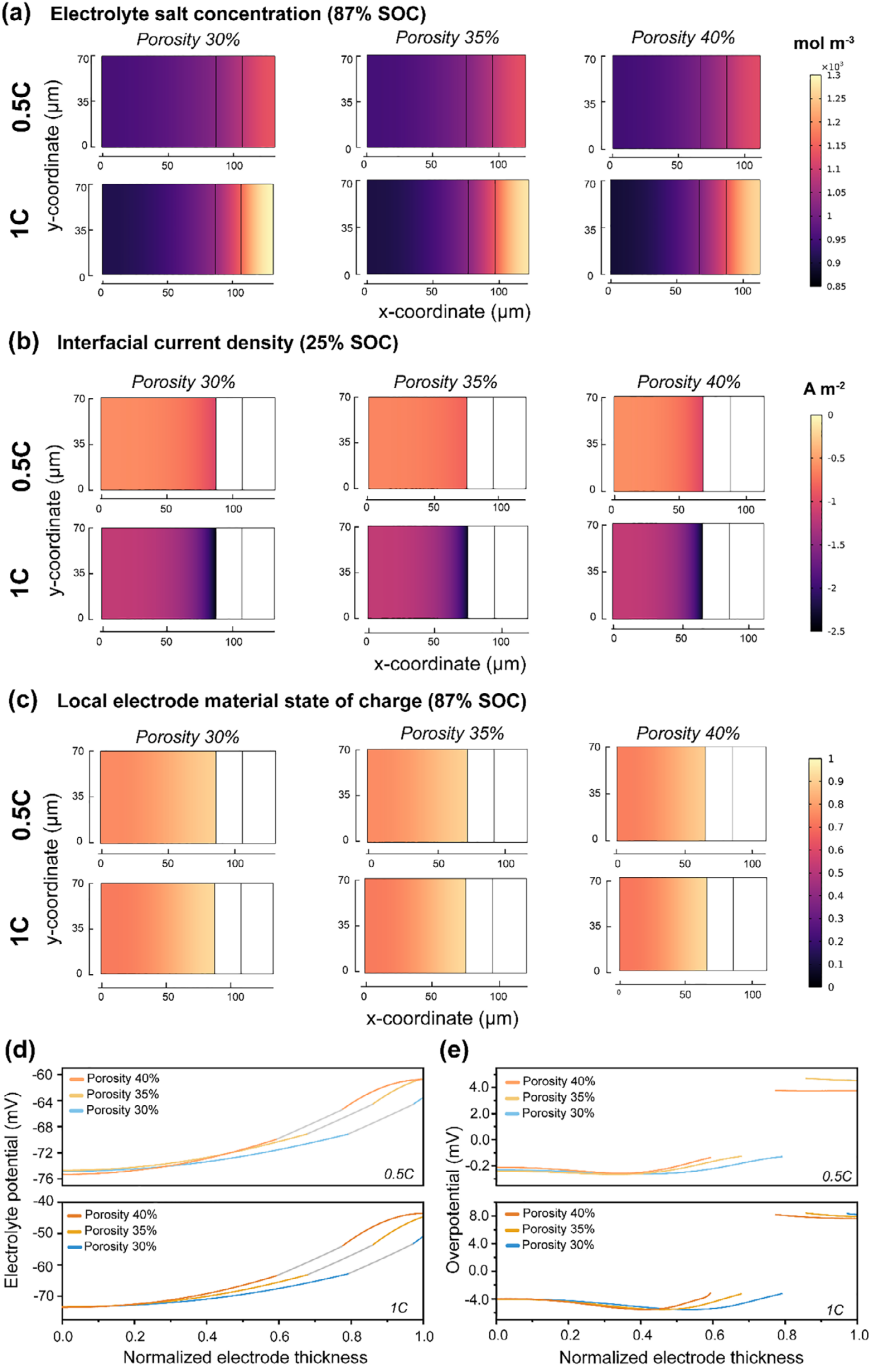
Continuum electrode‐scale COMSOL simulation of the transport‐induced origin of through‐thickness lithiation heterogeneity. Two‐dimensional spatial distributions comparing negative‐electrode porosities of 30%, 35%, and 40% at 0.5C and 1C for (a) Electrolyte salt concentration (c_l_) at late lithiation (87% SOC), (b) Local interfacial reaction current density at early lithiation (25% SOC) within the anode domain, showing severe reaction localization near the separator face at 1C, and (c) Local particle‐surface state of charge (SOC) at late lithiation (87% SOC) within the anode domain. Corresponding one‐dimensional profiles along the normalized electrode thickness at late lithiation show (d) Through‐thickness electrolyte potential profiles, in which the distinct grey curve tracks the potential within the separator domain (87% SOC), and (e) Local interfacial overpotential across the active electrode (87% SOC). Both the electrolyte potential and local interfacial overpotential profile enable direct comparison of the porosity‐dependent potential and overpotential variation under 0.5C and 1C conditions.

The operando‐OM observations in Section 2.2 demonstrate that decreasing electrode porosity promotes pronounced through‐thickness lithiation heterogeneity during elevated‐rate charging. These observations visualize the spatial evolution of graphite staging directly, but they do not reveal the electrochemical transport dynamics responsible for it. To identify the governing mechanism, continuum electrode‐scale simulations were performed using the Lithium‐Ion Battery interface of COMSOL Multiphysics [31, 32]. Two‐dimensional full‐cell models comprising a graphite negative electrode, a separator, a positive electrode, and current collectors were evaluated across systematic variations in negative‐electrode porosity (40%, 35%, and 30%) at 0.5C and 1C [33]. All other components, material properties, and charging conditions were held identical, so that the influence of porosity on transport and reaction behavior could be isolated. The full computational methodology, governing equations, boundary conditions, and parameters are provided in the Supporting Information. To capture liquid‐phase ionic transport, spatial distributions of the electrolyte salt concentration (c_l_) were evaluated across the full‐cell geometry at 87% SOC (Figure 3a and Figure S7). Under 0.5C, all three architectures exhibit relatively mild concentration gradients. Under 1C, however, severe liquid‐phase concentration polarization develops as porosity decreases. In the 40% porosity electrode, salt depletion near the anode current collector remains shallow (∼950 mol m^−^^3^); as porosity is reduced to 35%, moderate salt depletion emerges, signaling the onset of transport restriction. This behavior culminates in the 30% porosity electrode, which undergoes extreme depletion, dropping below 900 mol m^−^^3^ across the bulk interior of the anode (Figure 3a). Choked ionic transport within the low‐porosity, tortuous pore network therefore restricts Li^+^ replenishment to the electrode interior during elevated‐rate charging.

At 1C, decreasing electrode porosity is associated with increasingly pronounced spatial variations in electrolyte concentration and potential across the electrode thickness, accompanied by greater spatial variation in the local interfacial current density and overpotential. This progressive transport limitation on reaction kinetics is reflected directly in the local interfacial reaction current density during early lithiation at 25% SOC (Figure 3b and Figure S8). At 0.5C, the current‐density profiles remain spatially uniform across all porosities. Under 1C, the 40% porosity electrode maintains a relatively uniform reaction profile, whereas the 35% porosity electrode acts as a key intermediate state, with reaction activity beginning to shift noticeably towards the separator face. As porosity decreases further to 30%, this trend intensifies into a severe spatial reallocation of reaction activity: the intercalation flux concentrates into a sharp spike adjacent to the separator interface, reaching −2.5 A m^−^^2^, while the back of the electrode (x = 0 µm) remains underutilized (Figure 3b). This reaction localization dictates the spatial evolution of the local particle‐surface SOC during late lithiation (Figure 3c and Figure S9). The 40% porosity electrode maintains a coherent, relatively uniform lithiation front throughout its thickness, whereas the 35% porosity electrode develops a clear surface‐SOC gradient. In the 30% porosity electrode, this gradient reaches its extreme limit, driving premature local surface saturation at the separator interface (Figure 3c). Once particle surfaces near the separator reach thermodynamic saturation (LiC_6_), local intercalation kinetics shut down, forcing the active reaction zone deeper into the electrode. This produces the severe through‐thickness staging heterogeneity resolved in the operando‐OM depth profiles (Figure 2d).

The thermodynamic driving forces behind this heterogeneity, and the degradation that follows, were further quantified through liquid‐phase potential (ϕ_l_) and local interfacial overpotential (η) line profiles evaluated across the normalized electrode thickness (Figure 3d,e and Figures S10 and S11). The liquid‐phase ohmic drop steepens systematically as porosity decreases from 40% to 35% to 30% under 1C, owing to heightened ionic conduction resistance (Figure 3d), and the local interfacial overpotential consequently exhibits pronounced spatial variation. Under 1C (Figure 3e), the overpotential in the 30% porosity electrode shows the deepest negative excursion, dipping to approximately −5.5 mV and remaining highly polarized over a broader depth range than the 40% baseline. This localized negative overpotential, coupled with the premature surface saturation in the same region, establishes the thermodynamic driving force required for localized metallic lithium deposition. Taken together, these electrode‐scale simulations show that reduced porosity progressively chokes liquid‐phase ionic transport, with the 35% porosity electrode marking the onset at which reaction heterogeneity begins to develop actively. At lower porosity, these transport limitations intensify electrolyte‐potential drops, localize reaction flux near the separator, and drive premature local surface saturation. Together, these coupled mechanisms provide a framework that explains the transport‐induced through‐thickness staging heterogeneity and preferential lithium plating observed experimentally. The multiscale evolution of this heterogeneity is examined in the following section through analysis of graphite staging across both the electrode‐depth and in‐plane directions.

### 2.4 Hierarchical Development of Staging Heterogeneity From Individual Particles to the Electrode Plane

**FIGURE 4:**
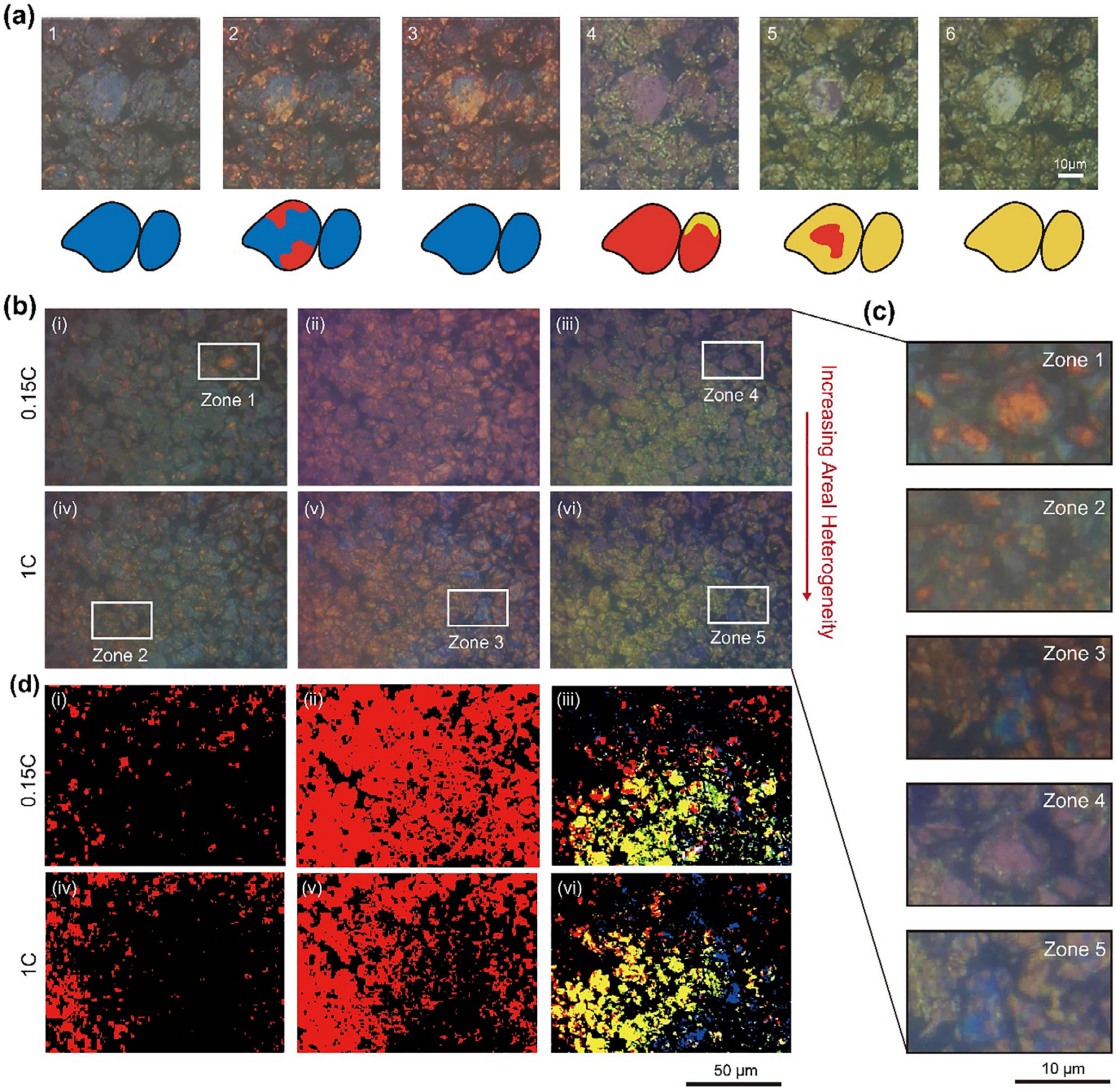
Areal (in‐plane) staging heterogeneity across the electrode surface and its intensification with charging rate. (a) Representative operando‐OM images of the sequential graphite staging evolution within individual particles during lithiation, with corresponding schematics illustrating the propagation of lithiation fronts. (b) Operando‐OM images comparing the 40% porosity electrode surface under low‐rate (0.15C) and high‐rate (1C) charging. The selected images correspond approximately to SOC values of 30%, 50%, and 60% at 0.15C and 10%, 18%, and 25% at 1C, respectively, showing the development of pronounced areal staging heterogeneity at the higher charging rate. (c) Magnified views of representative regions (Zones 1–5), highlighting the coexistence of different graphite staging states among neighboring particles under 1C charging. (d) Color extraction and image segmentation of individual staging phases using CIE L*a*b* color‐space analysis, mapping the spatial distribution of graphite stages and the increasing degree of areal heterogeneity with charging rate. The SOC values are approximate estimates under the operando‐OM measurement conditions.

The electrode‐scale simulations showed that reducing porosity intensifies transport limitation and drives pronounced through‐thickness lithiation gradients during elevated‐rate charging. These simulations explain the origin of the depth‐dependent heterogeneity, yet the operando‐OM results indicate that the spatial complexity of graphite lithiation extends beyond the through‐thickness direction. To resolve this, the lithiation process was examined across successive spatial scales, from individual graphite particles to the full electrode plane. Operando‐OM revealed that lithiation is non‐uniform even within individual particles. Rather than advancing uniformly, it preferentially initiates at localized regions and then propagates across the particle as charging proceeds, indicating spatially heterogeneous local reaction activity [34]. The behavior also varied considerably from particle to particle, reflecting differences in local reaction environment as shown in Figure 4a, some particles exhibited continuous phase‐front propagation, whereas others followed highly asymmetric or fragmented lithiation pathways. The propagation differed even between successive staging transitions within the same particle, so that distinct staging reactions frequently traced different trajectories. Graphite lithiation therefore cannot be described by a single diffusion‐controlled process or by charge transfer occurring uniformly over the particle surface; instead, the lithiation pathway evolves dynamically as the reaction proceeds, reflecting the changing kinetic and thermodynamic character of each staging transition, an effect that grows increasingly consequential under fast charging.

Having established that lithiation is already heterogeneous at the particle level, we next examined how this heterogeneity develops across the electrode surface under low‐ and high‐rate charging. Under low‐rate lithiation, the surface exhibited relatively uniform color evolution (Figure 4b,i‐iii), indicating that most regions underwent similar staging transitions throughout charging. Under 1C rate, the behavior changed markedly: Figure 4b‐iv‐vi) shows the simultaneous coexistence of blue, red‐orange, and golden‐yellow graphite phases within a single field of view. The presence of multiple staging states across neighboring regions demonstrates that lithiation no longer proceeds uniformly across the surface but becomes increasingly localized as the charging rate rises. This in‐plane heterogeneity becomes clearer on closer examination of representative surface regions (Figure 4c). Zone 1 contains predominantly blue and red‐orange phases, indicating that neighboring regions remain at distinctly different stages of lithiation. In Zone 2, localized golden‐yellow phase domains appear alongside the blue and red‐orange phases, showing that certain regions reach more advanced staging states considerably earlier than their surroundings. This contrast intensifies in Zone 3, where multiple staging states coexist over a larger fraction of the surface. The comparison between Zones 4 and 5 reinforces the trend: Zone 4 consists mainly of red‐orange and golden‐yellow phases, whereas Zone 5 retains a substantial fraction of the blue phase, confirming that pronounced lithiation differences persist even between adjacent regions during elevated‐rate charging. To map the spatial distribution of the individual staging states, color extraction and contrast enhancement were applied to the blue, red‐orange, and golden‐yellow phases. The processed images (Figure 4d) show that phase segregation becomes progressively more pronounced with charging rate: rather than evolving uniformly, lithiation localizes into well‐defined staging domains with markedly different lithiation states. The observed in‐plane reaction anisotropy can be attributed to the combined effects of electrode‐scale transport limitations and local microstructural variations across the porous graphite network. As demonstrated by continuum full‐cell simulations, reducing electrode porosity increases ionic transport resistance through the liquid electrolyte, steeping liquid‐phase potential gradients (Figure 3d) and reallocating interfacial reaction current towards the separator face under 1C rate (Figure 3b). This kinetic redistribution drives pronounced local variations in particle‐surface SOC (Figure 3c). Although the simulations represent a full‐cell configuration, these results provide electrode‐scale evidence for the development of spatially non‐uniform electrochemical conditions under reduced porosity. At the electrode surface in operando‐OM half‐cells, local variations in particle packing density, pore tortuosity, and electronic percolation pathways further modulate local Li^+^ flux and charge‐transfer rate among neighboring regions. As graphite lithiation progresses through discrete staging transitions, these local disparities cause neighboring domains to advance through the staging sequence asynchronously, resulting in coexistence of Stage 3L, Stage 2, and Stage 1 phases across the electrode surface as mapped by operando‐OM and CIE L*a*b* color analysis (Figure 4).

Collectively, these observations show that graphite staging heterogeneity develops hierarchically across spatial scales, becoming localized first within individual particles and then across the electrode surface to produce pronounced in‐plane heterogeneity during 1C charging. Combined with the through‐thickness gradients identified in Section 2.2, these results establish that reaction heterogeneity evolves simultaneously in both the depth and in‐plane directions of the porous electrode, completing the depth‐to‐areal picture of staging heterogeneity. Given the distinct local electrochemical environments across these domains, we sought to determine whether spatial staging heterogeneity directly triggers lithium plating, as explored in the following section.

### 2.5 Staging‐Driven Preferential Lithium Plating During Elevated‐Rate Charging

**FIGURE 5:**
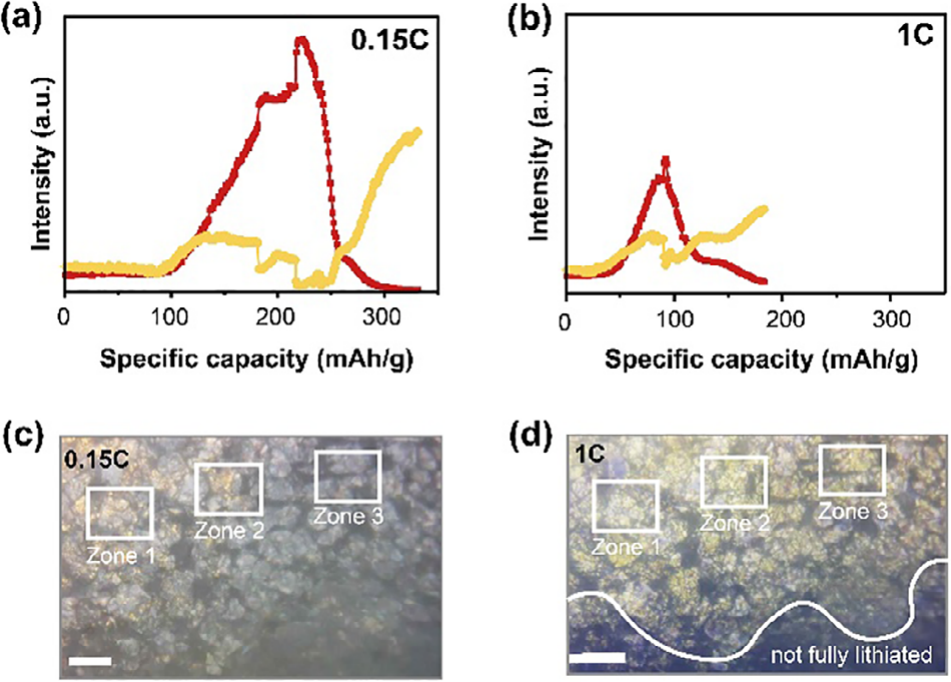
Temporal evolution of graphite staging phases revealing rate‐dependent areal heterogeneity. (a, b) Time evolution of the red–orange and golden‐yellow phases during lithiation at (a) 0.15C and (b) 1C. The fitted intensities represent the relative fractions of the intermediate (red‐orange) and advanced (golden‐yellow) staging phases extracted from operando‐OM images. (c, d) Corresponding operando‐OM images acquired at (c) 0.15C and (d) 1C. Representative regions (Zones 1–3) were selected for analysis; the incompletely lithiated region observed at 1C highlights the development of localized staging heterogeneity under elevated‐rate charging.

To connect graphite staging evolution to lithium plating, the temporal evolution of staging was tracked by operando‐OM combined with CIE L*a*b* color‐space analysis. The red‐orange and golden‐yellow optical components were extracted by color fitting, with the fitted intensity representing the relative fraction of each staging phase within the selected region, obtained from the surface area occupied by each color and fitted over time to follow the propagation of individual staging transitions. The red‐orange phase corresponds to intermediate staging, and the golden‐ yellow phase to more advanced lithiation states [16]. The evolution of the two phases at 0.15C and 1C is shown in Figure 5a,b. At 0.15C, the red‐orange phase rises gradually before transforming into the golden‐yellow phase, reflecting relatively continuous staging evolution. At 1C, by contrast, the maximum red‐orange phase intensity is markedly lower, and the golden‐yellow phase appears earlier, indicating that different regions advance through the staging sequence at different rates during elevated‐rate charging. The transition from intermediate to advanced stages thus becomes increasingly asynchronous as the charging rate rises.

The corresponding operando‐OM images support this picture (Figure 5c,d). At 0.15C, the selected regions (Zones 1–3) shared similar optical characteristics, indicating relatively uniform lithiation across the surface. At 1C, pronounced staging heterogeneity emerged, with localized golden‐yellow phase domains coexisting alongside neighboring regions still in earlier staging states; parts of the electrode remained incompletely lithiated even after prolonged charging. This behavior indicates that transport limitation suppresses uniform lithiation across the porous electrode and promotes increasingly localized reaction pathways during elevated‐rate charging.

This heterogeneous staging distribution points to a close relationship between localized advanced lithiation and the subsequent onset of lithium plating. Under 1C rate, the regions reaching the advanced golden‐yellow stage become increasingly localized while adjacent domains remain less lithiated. Operando observation of the continued lithiation reveals that lithium plating develops preferentially within these highly lithiated domains rather than uniformly across the surface (Figure S12). This spatial correspondence identifies localized advanced staging as the preferential site for plating initiation. A plausible origin is that highly lithiated graphite accommodates additional incoming lithium less readily than its less‐lithiated surroundings, so that further charge is increasingly diverted into metallic deposition at these sites [23, 34]; as in preferential nucleation during lithium‐metal growth, the advanced domains then serve as favorable sites for plating initiation and growth. Taken together with the through‐thickness gradients and multiscale staging heterogeneity established in the preceding sections, these results indicate that plating is not an isolated surface event but a consequence of transport‐induced reaction heterogeneity that builds up progressively during elevated‐rate charging.

Collectively, the operando‐OM observations link graphite staging heterogeneity to preferential lithium plating. Reduced porosity first intensifies transport limitation, generating through‐thickness lithiation gradients that evolve into pronounced in‐plane staging heterogeneity across the electrode surface; the resulting advanced‐stage domains then become the preferred nucleation sites for plating. Graphite staging heterogeneity is therefore not merely a consequence of fast charging but a precursor that governs where lithium plating initiates. This depth‐to‐areal framework clarifies how heterogeneous staging drives plating‐related degradation in graphite electrodes and identifies control of reaction heterogeneity, through rational electrode design, as a direct route to suppressing rate‐induced plating.

## 3 Conclusions

In this work, we resolved the origin and multiscale evolution of graphite staging heterogeneity during elevated‐rate charging and established it as a direct precursor to lithium plating. By combining electrochemical characterization, operando optical microscopy, in situ XRD, color‐space analysis, and electrode‐scale simulation, we tracked how a single control variable, electrode porosity, propagates through the electrode to shape where lithium ultimately plates. The central finding is that lithium plating is spatially templated by pre‐existing staging heterogeneity rather than nucleating at random. Operando imaging directly resolved staging heterogeneity developing across two coupled directions: through‐thickness (depth) gradients imposed by tortuosity‐controlled Li^+^ transport, and in‐plane (areal) non‐uniformity that emerges first within individual particles and then across the electrode surface. Crucially, we observed that the regions reaching advanced lithiation states earliest become the preferential sites at which lithium plating initiates. This depth‐to‐areal staging heterogeneity, visualized directly and in real time, links electrode architecture to plating onset through an unbroken chain: reduced porosity intensifies transport limitation, transport limitation drives asynchronous staging, and asynchronous staging templates the location of plating.

These findings reframe lithium plating during elevated‐rate charging. Rather than an isolated surface instability, plating is the terminal expression of reaction heterogeneity that accumulates progressively through the electrode, meaning that its location, and therefore its onset, is set well before metallic lithium appears. The advanced‐stage domains that seed plating are not stochastic defects but predictable outcomes of the interplay between electrode structure and transport. This mechanistic picture identifies electrode architecture as a direct design lever for suppressing rate‐induced plating. Because staging heterogeneity originates in tortuosity‐controlled transport, engineering the pore network to homogenize Li^+^ supply, through porosity, tortuosity, and their spatial distribution, offers a route to delay plating onset without sacrificing energy density. More broadly, the operando framework demonstrated here, correlating spatially resolved staging with plating in real time, provides a general tool for diagnosing heterogeneity‐driven degradation across graphite and other insertion electrodes, and for guiding the electrode designs required to make fast charging both faster and safer.

## 4 Experimental Section/Methods

### 4.1 Electrode Fabrication

Graphite electrodes were fabricated by slurry casting using spherical graphite (average particle size: 17.73± 7.11 µm) as the active material, Super P (Timcal) as the conductive additive, and sodium carboxymethyl cellulose (CMC) and styrene‐butadiene rubber (SBR) as binders. The electrode slurry, consisting of 96 wt.% graphite, 2 wt.% Super P, and 2 wt.% binder, was prepared using a Thinky mixer and coated onto a copper current collector using a doctor blade. The coated electrodes were dried at 80°C, calendered by roll pressing to obtain electrode porosities of 40%, 35%, 30%, and 20%, and subsequently vacuum‐dried at 110°C for 4 h. Circular electrodes (14 mm diameter) were punched for CR2032 coin‐type cell assembly. The graphite electrode was prepared with an areal capacity of approximately 3.5 mAh cm^−2^ for electrochemical characterization and operando optical microscopy experiments.

### 4.2 Electrochemical Characterization

Electrochemical measurements were performed using CR2032 coin‐type half‐cells assembled in an argon‐filled glove box with lithium metal as both the counter and reference electrode, a polyethylene (PE) separator (Celgard), and 1.3 M LiPF_6_ in EC:DEC (3:7 vol%) containing 10 wt.% fluoroethylene carbonate (FEC, PuriEL) as the electrolyte. Galvanostatic charge‐discharge measurements were carried out over a voltage range of 0.01–1.0 V. Prior to cycling, the cells underwent two formation cycles at 0.1C, followed by cycling at 0.2C. Rate capability was evaluated by sequential charging at 0.1, 0.2, 0.5, 1, 3, and 5C. Elevated‐charging performance was evaluated under a charging rate of 1C unless otherwise specified. All electrochemical measurements were conducted at room temperature using a battery cycler.

### 4.3 Electrode Morphology and Porosity Characterization

Cross‐sectional scanning electron microscopy (SEM) images of the calendered graphite electrodes were acquired at an accelerating voltage of 10 kV. The SEM images were analysed using ImageJ to generate binary segmentation maps of the pore and void regions and to visualize the changes in the electrode microstructure with increasing calendaring. Mercury intrusion porosimetry (MIP) was additionally performed to quantitatively characterize the porosity and pore‐size distribution of the electrodes.

### 4.4 Operando Optical Microscopy and Image Analysis

Operando‐OM was performed using an optical microscope (Olympus BX53M) coupled with a side‐by‐side electrochemical cell (EL‐CELL, ECC‐Opto‐SBS). The cell was assembled in an argon‐filled glovebox using the prepared graphite electrode as the working electrode, lithium metal as both the counter and reference electrode, and 1.3 M LiPF_6_ in EC:DEC (3:7 vol%) containing 10 wt.% fluoroethylene carbonate (FEC, PuriEL) as the electrolyte. Operando observations were conducted under 1C representative elevated‐rate charging and 0.5C reference charging conditions at 200x magnification. The evolution of graphite staging was quantitatively analysed using the LAB color space to monitor the spatial and temporal evolution of lithiation during charging.

### 4.5 In Situ X‐Ray Diffraction

In situ XRD measurements were performed using a SmartLab 9 kW diffractometer (Rigaku) equipped with a Cu Kα x‐ray source (λ = 1.541862 Å) and a D/teX Ultra 250 detector. Measurements were conducted in transmission geometry using a home‐made coin‐type cell modified from a standard CR2032 coin cell configuration. A central hole was introduced in the cap, spacer, and bottom case to allow the x‐ray beam to pass through the entire cell, and the holes in the cap and bottom case were sealed with a Kapton film to serve as an x‐ray transparent window while maintaining the airtightness of the cell. Successive diffraction scans were collected over a 2θ range of 23.5°–27°, covering the characteristic reflections associated with the graphite staging transitions, at a scan rate of 2°/min throughout the charging process, allowing continuous monitoring of the structural evolution of graphite during lithiation. The cell was subjected to the same fast‐charging protocol used for electrochemical characterization, cycled galvanostatically at 1C over a voltage range of 0.01–1.0 V, allowing the diffraction patterns to be directly correlated with the corresponding electrochemical profile.

### 4.6 Computational Details

A two‐dimensional full‐cell electrochemical model was developed using the Lithium‐Ion Battery interface in COMSOL Multiphysics V6.2 (COMSOL AB, Stockholm, Sweden) to investigate the influence of graphite electrode porosity on lithium transport and electrochemical reaction heterogeneity during elevated‐rate charging. The computational domain consisted of a graphite negative electrode, separator, NMC111 positive electrode, and corresponding current collectors. The graphite electrode was systematically varied (40%, 35%, and 30%), while all remaining material properties and operating conditions were kept constant. Simulations were performed under galvanostatic current conditions using a Current Distribution Initialization step followed by a time‐dependent study. The governing equation included charge conservation, electrolyte transport, solid‐state lithium diffusion, and Butler–Volmer interfacial kinetics. Detailed computational methodology, governing equations, numerical implementation, and simulation parameters are provided in the Supporting Information.

## Author Contributions

**Tae Young Choi**: conceptualization, methodology, investigation, Writing – original draft, Writing – review and editing, formal analysis, validation, visualization, data curation. **Juyoung Kim**: conceptualization, investigation, methodology, formal analysis, validation, visualization, writing – review and editing, data curation. **Vithiya Muralidharan**: visualization, writing – original draft, writing – review and editing, software, investigation, data curation. **Seungwoo Choi**: validation, visualization, formal analysis, writing – review and editing, investigation. **Jeongwoo Seo**: validation, visualization, formal analysis, writing – review and editing, investigation. **Seungwoo Ryu**: visualization, validation, writing – review and editing, formal analysis, investigation. **Myeongjun Choi**: writing – review and editing, visualization, formal analysis, investigation. **Hyun‐Wook Lee**: conceptualization, writing – review and editing, writing – original draft, funding acquisition, supervision, investigation.

## Conflicts of Interest

The authors declare no conflicts of interest

## Supporting information




**Supporting File**: advs77918‐sup‐0001‐SuppMat.docx.

## Data Availability

The data that support the findings of this study are available from the corresponding author upon reasonable request.
