# Supplementary material for: Depth‐to‐Areal Staging Heterogeneity Preceding Lithium Plating in Graphite Anodes Revealed by Operando Imaging
Source: Adv Sci (Weinh). 2026 Sep 27:e77918. Online ahead of print. doi: 10.1002/advs.77918 (PMC13616363; doi:10.1002/advs.77918)
Supplement: Supplementary file 1 [file ADVS-9999-e77918-s001.docx]

Supporting Information

Depth-to-Areal Staging Heterogeneity Preceding Lithium Plating in Graphite Anodes Revealed by Operando Imaging

Tae Young Choi^1,2^, Juyoung Kim^1,2^, Vithiya Muralidharan^1^, Seungwoo Choi^1^, Jeongwoo Seo^1^, Seungwoo Ryu^1^, Myeongjun Choi^1^, and Hyun-Wook Lee^1,^*

[1]	T.Y. Choi, J. Kim, V. Muralidharan, S. Choi, J. Seo, S. Ryu, M. Choi, H. -W. Lee

School of Energy and Chemical Engineering,

Ulsan National Institute of Science and Technology (UNIST),

Ulsan 44919, Republic of Korea

E-mail: [hyunwooklee@unist.ac.kr](mailto:hyunwooklee@unist.ac.kr) (H.-W. Lee)
[2]	T. Y. Choi and J. Kim contributed equally to this work.

Computational Details:

*Model Description*: A two-dimensional full-cell electrochemical model was developed using the Lithium-Ion Battery interface in COMSOL Multiphysics^®^ v6.2 (COMSOL AB, Stockholm, Sweden) to investigate the influence of graphite electrode porosity on electrochemical transport and lithiation heterogeneity during fast charging.^[1]^ The computational domain consisted of a graphite negative electrode, separator, LiNi_0.33_Mn_0.33_Co_0.33_O_2_ positive electrode, and the corresponding negative and positive current collectors, representing the full-cell configuration employed in this work. The electrolyte was modelled as LiPF_6_ dissolved in an EC:EMC (3:7) solvent mixture, while the graphite (Li_x_C_6_ MCMB) and NMC111 electrode properties were adopted from the COMSOL material library. To evaluate the influence of electrode architecture on electrochemical transport, the porosity of the graphite negative electrode was systematically varied to 40%, 35%, and 30%, whereas the separator and positive electrode properties were maintained unchanged.^[2]^ The initial state of charge (SOC) of the full cell was set to 5%, and the initial lithium distribution between the positive and negative electrode was established using the build in SOC and Initial Charge Distribution feature of the Lithium-Ion Battery interface.^[3]^

The porous electrodes were modelled using the porous electrode formulation coupled with lithium intercalation within spherical active-material particles. Charge transport in the electronic and electrolyte phases, lithium-ion transport in the electrolyte, lithium diffusion within the active material particles, and interfacial electrochemical reaction were solved simultaneously using the Multiphysics formulation implemented in the Lithium-Ion Battery interface. The simulations were performed under galvanostatic current conditions using a Current Distribution Initialization step followed by a time-dependent study. Current Distribution Initialization was employed to establish a stable initial potential distribution before solving the fully coupled transient electrochemical transport properties. The transient charging process was subsequently simulated using an implicit time-stepping scheme together with indicator states and implicit event handling, allowing the simulation to terminate automatically once the given cutoff voltage was reached. A parametric sweep was performed to investigate different charging rates, while the charging conditions discussed in the manuscript correspond to 0.5 C and 1 C. The computational model was used to obtain the spatial distributions of electrolyte concentration, lithium-ion flux, electrolyte current density, local reaction current density, electrolyte potential, interface overpotential, and state of charge during charging. These variables were subsequently analysed to establish the relationship between electrode porosity, electrochemical transport limitations, and the experimentally observed through-thickness lithiation heterogeneity.

Although half-cell configurations were utilized for operando characterization, a full cell computational geometry (Graphite || NMC111) was implemented in COMSOL to accurately simulate the dynamic mass transport, salt depletion, and liquid phase potential gradients across the complete inter-electrode domain under galvanostatic fast charging.

***Governing Equations***: The electrochemical behaviour of the full-cell model was described using porous-electrode theory, which simultaneously considers electronic charge transport in the solid phase, ionic transport in the electrolyte, lithium diffusion within the active material particles, and interfacial electrochemical reactions. The governing equations implemented in the Lithium-Ion Battery interface are summarized below.

Electronic charge conservation

Charge transport in the electronically conductive phase of the porous electrodes is governed by Ohm’s law coupled with charge conservation:

$\nabla\cdot i_{s}= -i_{v,tot}$	(S1)

Where $i_{s}$ is the electronic current density in the solid phase and $i_{v,tot}$ is the volumetric interfacial reaction current density.

The electronic current density is expressed as

$i_{s}= -\sigma_{s,eff}\nabla\phi_{s}$	(S2)

Where $\sigma_{s,eff}$ is the effective electronic conductivity and $\phi_{s}$ is the solid-phase potential.

Electrolyte charge conservation

Charge conservation within the electrolyte phase is expressed as

$\nabla\cdot i_{l}= -i_{v,tot}$	(S3)

Where $i_{l}$ is the electrolyte current density.

The electronic current density is expressed as

$i_{l}= -\kappa_{\mathrm{eff}}\nabla\phi_{l}+ \frac{2\kappa_{\mathrm{eff}}\mathrm{RT}}{F}\left( 1+ \frac{\partial\ln f}{\partial\ln c_{1}} \right)\left( 1-t_{+} \right)\nabla\ln c_{1}$	(S4)

Where $\kappa_{\mathrm{eff}}$ is the effective electrolyte conductivity, $\phi_{l}$ is the electrolyte potential, $c_{1}$ is the electrolyte concentration, $t_{+}$ is the lithium-ion transference number, $R$ is the universal gas constant, $T$ is the absolute temperature and $F$ is the Faraday’s constant.

Lithium-ion transport in the electrolyte

Lithium-ion transport within the electrolyte obeys species conservation

$\frac{\partial\left( \varepsilon c_{1} \right)}{\partial t}= -\nabla\cdot N_{l}+\frac{1-t_{+}}{F}i_{v,tot}$	(S5)

Where $\varepsilon$ is the electrolyte volume fraction and $N_{l}$ is the lithium-ion flux.

The lithium-ion flux is given by

$N_{l}=-D_{\mathrm{eff}}\nabla c_{1}+\frac{t_{+}}{F}i_{l}$	(S6)

Where $D_{\mathrm{eff}}$ is the effective electrolyte diffusion coefficient.

Lithium diffusion within active material particles

Lithium transport inside the spherical graphite and NMC111 particles follows Fick’s second law

$\frac{\partial c_{s}}{\partial t}=\frac{1}{r^{2}}\frac{\partial}{\partial r}\left( D_{s}r^{2}\frac{\partial c_{s}}{\partial r} \right)$	(S7)

Where $c_{s}$ is the lithium concentration in the solid particle, $D_{s}$ is the solid-state diffusion coefficient and $r$ is the radial coordinate within the particle.

Interfacial electrochemical reaction

The interfacial kinetics were described using the Butler-Volmer equation^[4]^

$i_{\mathrm{loc}}=i_{0}\left[ \exp\left( \frac{\alpha_{a}F\eta}{\mathrm{RT}} \right)-exp\left( -\frac{\alpha_{c}F\eta}{\mathrm{RT}} \right) \right]$	(S8)

Where $i_{0}$ is the exchange current density, $\alpha_{a}$ and $\alpha_{c}$ are the anodic and cathodic charge transfer coefficients, respectively, and $\eta$ is the interfacial overpotential.

The interfacial overpotential is defined as

$\eta=\phi_{s}{- \phi}_{l}-U$	(S9)

Where $U$ represents the equilibrium potential of the active material.

State-of-charge Initialization

The initial lithium distribution between the positive and negative electrodes was established using the built-in SOC and Initial Charge Distribution feature of the Lithium-Ion Battery interface. The cell capacity was calculated as

$\mathrm{Cap}_{\mathrm{cell}}=Q_{inv, neg}^{100\%SOC}-Q_{inv, neg}^{0\%SOC}$	(S10)

And the cell state of charge is determined by

$\mathrm{SOC}_{\mathrm{cell}}=\frac{Q_{inv, neg}-Q_{inv, neg}^{0\%SOC}}{\mathrm{Cap}_{\mathrm{cell}}}$	(S11)

Where $Q_{inv, neg}$ is the lithium inventory in the negative electrode.

The lithium inventory in each electrode was calculated from the volume-averaged lithium concentration within the porous active material, ensuring consistent initialization of the full-cell equilibrium before transient charging simulations.

Initial Lithium Inventory and SOC formulation

This procedure determines the initial lithium inventory in both electrodes by enforcing lithium conservation and electrochemical equilibrium before the transient charging simulation. The total host capacity of each electrode is calculated from the maximum lithiation concentration within the active material according to the

$Q_{host,neg}= \int_{\Omega_{\mathrm{neg}}} \varepsilon_{s}c_{s,max,neg}Fd\Omega$	(S12)

$Q_{host,pos}= \int_{\Omega_{\mathrm{pos}}} \varepsilon_{s}c_{s,max,pos}Fd\Omega$	(S13)

Where $Q_{\mathrm{host}}$ represents the theoretical lithium storage capacity of each porous electrode, $\varepsilon_{s}$ is the active material volume fraction, $c_{s,max}$ is the maximum lithium concentration in the solid phase, $F$ is the Faraday’s constant and $\Omega$ denotes the electrode domain.

The initial lithium inventory which each electrode is determined from the average lithium concentration inside the active material:

$Q_{inv,neg}= \int_{\Omega_{\mathrm{neg}}} \varepsilon_{s}c_{s,avg,neg}Fd\Omega$	(S14)

$Q_{inv,pos}= \int_{\Omega_{\mathrm{pos}}} \varepsilon_{s}c_{s,avg,pos}Fd\Omega$	(S15)

Where $c_{s,avg}$ is the volume-averaged lithium concentration within the spherical active-material particles.

For a full-cell configuration, lithium conservation requires that the total lithium inventory remains constant throughout the initialization:

$Q_{inv,tot}= Q_{inv,neg}+Q_{inv, pos}=constant$	(S16)

The cell capacity is subsequently determined from the lithium inventory corresponding to the fully discharged and fully charged equilibrium states using Equation S10. In the present study, the initial cell SOC was specified as 5%, and the corresponding equilibrium lithium distribution between the graphite negative electrode and the NMC111 positive electrode was automatically established through SOC initialization procedure before the transient galvanostatic charging.

**Figure S1. (a)** Cross-sectional SEM micrographs of graphite electrodes with nominal porosities of 40%, 30-35 % and 20-30% (scale bar = 50 μm). **(b)** Corresponding binary threshold segmentation maps generated via ImageJ, where black regions denote the interconnected void/pore network and white regions represent the solid active material matrix. **(c)** Log differential intrusion volume versus pore size diameter obtained from Mercury intrusion porosimetry (MIP), illustrating the reduction in dominant inter-particle macropore volume (~3-4 μm) and intra-network mesopores (~0.2 μm) upon calendaring. **(d)** Directional electrode tortuosity factor (τ) plotted as a function of electrode porosity, demonstrating the non-linear increase in ionic transport resistance at low porosity levels.

**Figure S2.** Galvanostatic charge-discharge voltage profiles of graphite electrodes with 40%, 35%, and 30% porosity measured at **(a)** 0.1 C and **(b)** 1 C. The voltage profiles are plotted as a function of normalized capacity to compare the influence of electrode porosity on electrochemical polarization under low and high-rate charging conditions.

**Figure S3.** Long term cycling performance of graphite electrodes with 40%, 30%, and 20% porosity during repeated charge-discharge cycling. **(a)** Specific capacity as a function of cycle number. **(b)** Constant-Voltage (CV) step capacity ratio during cycling for electrodes with different porosities.

The CV step capacity ratio was calculated using equation S17

$CV step capacity ratio \left( \% \right)= \left( \frac{Capacity delivered during CV step}{Total charge capacity} \right)\times100$	(S17)

**Figure S4.** Differential capacity (dQ/dV) curves of graphite electrodes with 40%, 30%, and 20% porosity measured at 0.1 C, 0.5 C, and 1 C. The lower panels show enlarged views of the low-voltage region to highlight changes in the graphite staging peaks with electrode porosity and charging rate.

**Figure S5.** In-Situ X-ray diffraction (XRD) patterns measured during lithiation of graphite electrodes with **(a)** 40% and **(b)** 30% porosity. The evolution of the graphite (002), LiC_12_ (002), and LiC_6_ (001) diffraction peaks are shown as a function of lithiation process, illustrating the structural phase transformation of graphite during lithiation intercalation.

**Figure S6.** Cross-sectional operando optical microscopy (OM) images of graphite electrodes with 40% and 30% porosity acquired during lithiation under 0.15 C charging conditions. **(a-d)** represent successive stages of lithiation, illustrating the evolution of graphite staging across the electrode thickness with different porosities.

**Figure S7.** Spatial distribution of electrolyte salt concentration (c_l_) across electrode thickness (x-coordinate) and height (y-coordinate) at varied SOCs (25%, 50%, and 75% SOC), charging rates (0.5C and 1C), and porosity (30%, 35%, and 40%). The color bar in the right indicates the local salt concentration in mol m^-3^.

**Figure S8.** Spatial distribution of local interfacial reaction current density across electrode thickness (x-coordinate) and height (y-coordinate) at varied SOCs (50%, 75%, and 87% SOC), charging rates (0.5C and 1C), and porosity (30%, 35%, and 40%). The color bar in the right indicates the interfacial current density in A m^-2^.

**Figure S9.** Spatial distribution of local particle surface state of charge across electrode thickness (x-coordinate) and height (y-coordinate) at varied SOCs (25%, 50%, and 75% SOC), charging rates (0.5C and 1C), and porosity (30%, 35%, and 40%). The color bar indicates the dimensionless stoichiometry ranging from 0 to 1.

**Figure S10.** Electrolyte potential distribution along the normalized electrode thickness under varying porosities **(a)** 30% **(b)** 35%, and **(c)** 40%. Each panel compares the potential profiles across different SOCs at the charging rates 0.5C (top panels) and 1C (bottom panels).

**Figure S11.** Reaction overpotential along the normalized electrode thickness under varying porosities **(a)** 30%, **(b)** 35%, and **(c)** 40%. Each panel compares the potential profiles across different SOCs at the charging rates 0.5C (top panels) and 1C (bottom panels).

**Figure S12.** Representative operando OM images showing the initiation and progression of lithium plating during high C-rate lithiation. The red circles indicate regions where lithium plating is observed, while the white outline highlights an incompletely lithiated region beneath the plated surface during later stage of lithiation.

**Table S1.** Summary of microstructural and porosimetric properties of the fabricated graphite electrodes across different calendaring states

| **Electrode designation** | **Target porosity**  **(%)** | **Measured MIP porosity**  **(%)** | **Bulk density**  **(g mL^-1^)** | **Mean pore diameter**  **(µm)** |
| --- | --- | --- | --- | --- |
| **High** | 40% | 40.1% | 2.17 | 0.68 |
| **Medium** | 35% | 35.1% | 2.15 | 0.63 |
| **Low** | 20-30% | 28.9% | 2.20 | 0.59 |

**REFERENCES**

[1]	COMSOL **2024**, *COMSOL Multiphysics*^®^ version **6.2**

[2]	E. Hosseinzadeh, J. Marco, P. Jennings, “The impact of multi-layered porosity distribution on the performance of a lithium ion battery” *Appl. Math. Model.* **2018**, *61*, 107–123.

[3]	L. Cai, R. E. White, “Mathematical modeling of a lithium ion battery with thermal effects in COMSOL Inc. Multiphysics (MP) software” *J. Power Sources* **2011**, *196*, 5985–5989.

[4]	M. Doyle, T. F. Fuller, J. Newmann, “Modeling of Galvanostatic Charge and Discharge of the Lithium/Polymer/Insertion Cell” *J. Electrochem. Soc* **1993**, *140*, 1526.
